# Choosing appropriate patient‐reported outcome measures for prostate disease

**DOI:** 10.1002/bco2.136

**Published:** 2022-01-12

**Authors:** Alexander Ng, Pramit Khetrapal, Chris Brew‐Graves, Nicola Muirhead, Aqua Asif, Vinson Wai‐Shun Chan, Arjun Nathan, Veeru Kasivisvanathan

**Affiliations:** ^1^ UCL Medical School University College London London UK; ^2^ British Urology Researchers in Surgical Training (BURST) London UK; ^3^ Division of Surgery and Interventional Science UCL London UK; ^4^ Department of Urology Whipps Cross Hospital, Barts Health NHS Trust London UK; ^5^ National Cancer Imaging Translational Accelerator (NCITA), Division of Medicine UCL London UK; ^6^ Leicester Medical School University of Leicester Leicester UK; ^7^ School of Medicine, Faculty of Medicine and Health University of Leeds Leeds UK

**Keywords:** approval, education, patient‐reported outcome measure, prostate, prostate disease

Validated patient‐reported outcome measures (PROMs) have an important role in clinical practice and research studies. The outcomes may be used for patient counselling, clinician decision‐making and disease or treatment monitoring. Critically, PROMs are an important outcome for comparison of efficacy of treatment approaches in research studies. However, PROMS remain under‐utilised in research. It is not uncommon for studies to be clinician‐led; thus, prioritisation of outcomes deemed more important to the clinician can lead to the omission of PROMS. Further challenges for clinicians exist in identifying relevant PROMs for their study and understanding the appropriate approvals required.^1^ Other challenges include ensuring high completion rates of questionnaires by patients. We describe our experience of choosing between different PROMs and obtaining relevant approvals within the scope of an interventional study of prostate cancer (NCT04571840). This article may encourage the use of PROMs in future research, aid clinicians and researchers in choosing the most appropriate PROM, and navigate the approval process. This will help researchers plan prospective patient‐centred research and account for possible approvals and costs in grant applications.

Our study context involved designing an interventional study evaluating two forms of MRI for the diagnosis of prostate cancer, standard of care multiparametric MRI and an abbreviated version, biparametric MRI, which omits the contrast sequence. If the abbreviated version is as good as the full multiparametric MRI, it might be a more cost‐effective diagnostic standard of care test for prostate cancer. Part of the evaluation of the pros and cons of shortening the scan include possible implications on diagnostic accuracy, staging and subsequently treatment decisions. Thus, evaluation of baseline continence, lower urinary tract symptoms and erectile function can be important to guide the multi‐disciplinary team decision‐making for treatment choice and monitoring and managing side effects of treatment. A summary of commonly used instruments that we considered for this study in prostate cancer are outlined in Table 1.

**TABLE 1:** The objective(s), copyright owner and cost for the use of five validated questionnaires in urological patients

	Number of items	What does it assess?	Validated population	Copyright/who issues approval/permission	Cost^a^		Article authors are required to reference in publication
IIEF‐5 (SHIM score) International Index of Erectile Function	5	Presence and severity of erectile dysfunction	Heterosexual men in stable relationships for ≥6 months who have had the opportunity to engage in sexual activity and intercourse	Pfizer Patient‐Centred Outcomes Assessment^2^	No cost		DOI: 10.1038/sj.ijir.3900472
I‐PSS International Prostate Symptom Score	8	Severity of urinary symptoms related to BPH	Men with BPH	Public domain^3^	No cost		N/A
ICIQ‐UI‐SF International Consultation on Incontinence Questionnaire—Urinary Incontinence Short Form	4	Symptoms and impact of urinary incontinence	Patients with incontinence or other lower urinary tract symptoms	The International Consultation on Incontinence Questionnaire^4^	Clinical (routine use)	No cost	N/A
					Research (small grants or student projects)	No cost	
					Research (large grants e.g. NIHR funded or equivalent)	£1000 per module of translation	
EQ‐5D‐3L EuroQoL 5‐Dimension 3‐Level	6	Health related quality of life for any patient with any pathology	Adults	EuroQoL^5^	No cost		N/A
EPIC‐26 Expanded Prostate Cancer Index Composite Short Form	26	Patient function and bother after prostate cancer treatment	Men with PCa	The University of Michigan^6^	No cost		DOI: 10.1016/s0090‐4295(00)00858‐x

Abbreviations: BPH, benign prostatic hyperplasia; ED, erectile dysfunction; PCa, prostate cancer.

^a^ Cost for non‐commercial, investigator‐led studies.

When choosing the right PROM, researchers must decide on whether to use a generic or specific questionnaire, that evaluate a range of clinical conditions (e.g., EQ‐5D‐3L), or at a specific disease, population and symptom (e.g., EPIC‐26), respectively. Whilst generic PROMs enable researchers to compare outcomes across different disease and symptom populations, specific PROMs do not. Therefore, both generic and specific PROMs can be used concurrently to combine the benefits of both. We considered that the use of generic health‐related quality of life as measured by EQ‐5D‐3L would have been of value if we had planned to randomise patients to one diagnostic test or another. However, as our study design was a within patient design, we felt that it would not be as valuable as it would not be possible to attribute the quality of life measurement as being from one test or the other since each patient gets both tests.

Another consideration is the length of the questionnaire. There have been developments of abridged versions of the same questionnaire. For example, in our study, we considered whether to use the full version International Index of Erectile Function (IIEF) questionnaire or whether to use to abridged version of this, the IIEF‐5, to evaluate the presence and severity of baseline erectile function. The shorter versions provide the benefits of being quicker to complete, and would encourage a greater response rate. Whilst shorter and abridged versions appear ideal, there is a trade‐off between time to complete and the amount of information collected. Therefore, it is important to consider the evidence supporting the validation of a PROM from the developers, to ensure that the abridged version is as accurate as the original questionnaire (e.g., the IIEF‐5 questionnaire demonstrates an area under the ROC curve of 0.97^7^). We felt that as the primary outcome of the study was not related to erectile function and was focussed on prostate cancer detection, the abridged IIEF‐5 version would suit the purpose which was to provide PROMs to inform research multi‐disciplinary team meeting decisions on patient treatment eligibility.

To reduce attrition bias, it is important to use the minimal number of essential PROMs questionnaires to address the study hypothesis. From our experience, this is best done by coordinating these with mandatory patient visits so that a reminder can be given directly to the patient if required. In our study, we did this by collecting PROMS at the mandatory baseline visit and carrying out patient consultations at the study design stage to ensure what we were proposing would be reasonable for a patient.

Other important considerations include the validation status of a PROM. This is key in determining its relevance for use on a population of interest. This is typically conducted via an external validation process to ensure accuracy. The key measurement properties assessed are reliability and validity.^1^ Reliability is assessed through internal consistency and reproducibility; whilst validity is assessed through face, content and construct validity as the most relevant measures for PROM instruments.^1^ Using a PROM for an unintended purpose, on a non‐target population, or without seeking the appropriate approvals risks undermining the validity of a study.

Non‐clinical considerations are equally important in selecting the right PROM. This includes seeking permission to use the PROM and any associated costs. Approvals are generally straightforward and can be obtained promptly, with some in the public domain, and others under academic institutions or commercial companies (Table 1). For international studies, acquiring translations can be an important factor to consider. English is the default language for many PROMs instruments, with validated translations sometimes incurring additional costs or delays. Where permission to translate into a language that is not yet available is sought, sometimes the institution or company will require the authors to forward the final renders, reports and associated certificates of translation. It is also important to consider the cost of administering the questionnaires, such as printing costs if providing hard copies to patients to complete, and time associated with inputting these data onto an electronic case report form. Both of these can be covered in research studies on a per patient basis as part of the grant application. However, in certain centres, the collection of PROM data may be standard of care and may therefore pose no additional time or financial burden.

We hope that the framework provided in this article can serve as a helpful starting point for incorporating PROMs into research studies with particular consideration for prostate cancer research. We would encourage their inclusion in interventional studies and advise researchers to ensure that they have appropriate patient and public involvement at the study design stage to help establish where they might be best used in a study. We would encourage researchers to use appropriately validated questionnaires, factor costs in for their use and have given examples of the types and means of obtaining approvals for some commonly used questionnaires in urology.

## CONFLICT OF INTEREST

The authors have no conflict of interest to declare.

## AUTHOR CONTRIBUTIONS

V. K. conceptualised the idea. A. N., P. K., A. A., V. W. S. C. and V. K. developed the concept. A. N. wrote the first draft of the manuscript. All authors were involved in editing, critical review and final approval of the manuscript.

## FUNDING INFORMATION

The PRIME Study (NCT04571840) is funded by Prostate Cancer UK, the John Black Charitable Trust Grant, the European Association of Urology Research Foundation and the Dieckmann Foundation.
